# 快速滤过型净化结合超高效液相色谱-串联质谱法测定中华绒螯蟹中14种全氟烷基化合物

**DOI:** 10.3724/SP.J.1123.2023.07017

**Published:** 2023-12-08

**Authors:** Xianli WANG, Qinxiong RAO, Qicai ZHANG, Penghui DU, Weiguo SONG

**Affiliations:** 上海市农业科学院农产品质量标准与检测技术研究所,上海市农产品质量安全评价技术服务平台,上海201403; Institute for Agro-food Standards and Testing Technology, Shanghai Academy of Agricultural Sciences, ShanghaiAgricultural Product Quality and Safety Evaluation Technical Service Platform, Shanghai 201403, China

**Keywords:** 快速滤过型净化, 超高效液相色谱-串联质谱, 全氟烷基化合物, 中华绒螯蟹, multi-plug filtration cleanup (m-PFC), ultra-performance liquid chromatography-tandem mass spectrometry (UPLC-MS/MS), perfluoroalkyl substances (PFASs), Chinese mitten crab

## Abstract

采用基于羧基多壁碳纳米管填料的快速滤过型净化柱(multi-plug filtration cleanup, m-PFC)消减样品中的脂肪和磷脂等杂质干扰,利用超高效液相色谱-串联质谱(UPLC-MS/MS)建立了同时测定中华绒螯蟹(*Eriocheir sinensis*)中14种全氟烷基化合物(perfluoroalkyl substances, PFASs)的方法。样品加入5 mL 0.1%甲酸水溶液涡旋混匀,接着加入15 mL乙腈,加入2 g无水硫酸钠和2 g氯化钠萃取盐,涡旋振荡3 min,离心后取10 mL上清液过快速滤过型净化柱,流出液氮吹浓缩近干,用甲醇复溶至1 mL后,取上清液过0.22 μm滤膜,采用Shimadzu Shim-pack G1ST-C18色谱柱(100 mm×2.1 mm, 2 μm)分离,以甲醇和5 mmol/L乙酸铵溶液体系为流动相进行梯度洗脱,在电喷雾负离子模式下,以多反应监测模式(MRM)采集数据,内标法定量分析。该研究优化了液相色谱条件,5 mmol/L乙酸铵溶液-甲醇体系作为流动相时14种PFASs普遍具有更佳的峰形和灵敏度。在最佳的实验条件下,14种PFASs在一定范围内呈良好线性,相关系数(*R*^2^)为0.998~0.999; 14种目标物质的方法检出限(LOD)为0.03~0.15 μg/kg,定量限(LOQ)为0.10~0.50 μg/kg; 3个添加水平下14种PFASs的平均回收率为73.1%~120%,相对标准偏差(RSD, *n*=6)为1.68%~19.5%。运用该方法对上海3个养殖基地的中华绒螯蟹样品进行定量检测。该方法简便、高效,大幅提升了检测效率,适用于中华绒螯蟹中14种PFASs的快速分析。

## Abstract (en)

Perfluoroalkyl substances (PFASs) have become a new food-safety problem. Dietary intake is a major pathway of human exposure to PFASs. Chinese mitten crab (*Eriocheir sinensis*) is a high-end aquaculture product popular among consumers in China. Conventional extraction methods for PFASs are cumbersome and time consuming, and result in incomplete purification; thus, this technique does not meet the requirements for PFAS detection. Herein, an analytical strategy was established for the rapid detection of 14 PFASs in Chinese mitten crab based on multi-plug filtration cleanup (m-PFC) and ultra-performance liquid chromatography-tandem mass spectrometry (UPLC-MS/MS). The carbon-chain length of the 14 PFASs analyzed in this study ranged from 4 to 14, and they are perfluorobutanoic acid (PFBA), perfluoro-*n*-pentanoic acid (PFPeA), perfluorohexanoic acid (PFHxA), perfluoroheptanoic acid (PFHpA), perfluorooctanoic acid (PFOA), perfluorononanoic acid (PFNA), perfluorodecanoic acid (PFDA), perfluoroundecanoic acid (PFUnDA), perfluorododecanoic acid (PFDoDA), perfluorotetradecanoic acid (PFTeDA), perfluoro-1-butane sulfonic acid (PFBS), perfluoro-1-hexane sulfonic acid (PFHxS), perfluoro-1-octane sulfonic acid (PFOS), and perfluoro-1-decanesulfonate (PFDS). The m-PFC column was prepared using carboxy-based multiwalled carbon nanotubes, and used to reduce the interference of sample impurities. The samples were extracted with 5 mL of 0.1% formic acid aqueous solution, 15 mL of acetonitrile and extraction salt (2 g Na_2_SO_4_ and 2 g NaCl). The supernatant (10 mL) was purified using the m-PFC column, concentrated to near dryness under nitrogen, and then redissolved in 1 mL of methanol. Finally, the sample solution was filtered through a 0.22 μm polypropylene syringe filter for UPLC-MS/MS analysis. The target analytes were separated using a Shimadzu Shim-pack G1ST-C18 chromatographic column (100 mm×2.1 mm, 2 μm) using methanol (A) and 5 mmol/L ammonium acetate aqueous solution (B) as the mobile phases via gradient elution. The linear gradient program were as follows: 0-0.5 min, 10%A-35%A; 0.5-3 min, 35%A-60%A; 3-5 min, 60%A-100%A; 5-6.5 min, 100%A; 6.5-7 min, 100%A-10%A. The target analytes were analyzed using negative electrospray ionization in multiple-reaction monitoring mode, and quantitative analysis was performed using the internal standard method. In this study, we optimized the mobile-phase system as well as the extraction solvent, time, volume, and salt. The 14 PFASs exhibited good peak shapes and sensitivities when the 5 mmol/L ammonium acetate solution-methanol system was used as the mobile phase. Compared with acetonitrile or methanol alone, the extraction efficiencies of the 14 PFASs were significantly improved when 5 mL of 0.1% formic acid aqueous solution was added, followed by 15 mL of acetonitrile. The extraction efficiencies of the 14 PFASs did not differ significantly when the extraction time was within 3-15 min. The extraction salt (MgSO_4_, Na_2_SO_4_, NaCl, (NH_4_)_2_SO_4_, and Na_2_SO_4_+NaCl) significantly affected the extraction efficiencies of the 14 PFASs. The highest extraction efficiencies of the 14 PFASs, which ranged from 47.9% to 121.9%, were obtained when Na_2_SO_4_+NaCl was used as the extraction salt. Under the optimal experimental conditions, good linearities (*R*^2^=0.998-0.999) were obtained for seven PFASs (PFBS, PFHxA, PFHpA, PFHxS, PFDA, PFDoDA, PFTeDA) at 0.10-100 μg/L, and seven PFASs (PFBA, PFPeA, PFOA, PFOS, PFNA, PFUnDA, PFDS) at 0.50-100 μg/L. The average spiked recoveries for the 14 PFASs in Chinese mitten crabs at three levels ranged from 73.1% to 120%, with relative standard deviations (RSDs) in the range of 1.68%-19.5%(*n*=6). The limits of detection (LODs) and quantification (LOQs) of the 14 PFASs were in the range of 0.03-0.15 and 0.10-0.50 μg/kg, respectively. The developed method was applied to the analysis of crab samples collected from three farms in Shanghai, and PFASs with total concentrations of 3.52-37.77 μg/kg were detected in all samples. The detection frequencies for PFDA, PFUnDA, PFDoDA, PFTeDA, and PFOS were 100%. PFDA, PFUnDA, PFOS, and PFDoDA were the most abundant congeners, accounting for 31.2%, 30.6%, 15.0%, and 10.9%, respectively, of the 14 PFASs detected. The proposed method is simple, efficient, accurate, and suitable for the rapid analysis of 14 PFASs in Chinese mitten crabs.

全氟烷基化合物(perfluoroalkyl substances, PFASs)是一类高度稳定的新型持久性有机污染物,由于独特的疏水疏油特性被广泛应用于化工、电镀、涂料、纺织、皮革、合成洗涤剂、炊具制造和消防设施等诸多领域^[1,2]^。PFASs难以被物理、化学及生物作用降解,甚至在某些强氧化剂和强酸碱等极端条件下仍可以保持稳定,目前在环境和生物体中被广泛检出^[3]^。PFASs具有高毒、持久、长距离迁移及随食物链蓄积放大的特性,毒理学研究发现其对内分泌、神经、免疫、生殖等系统均具有毒性和致癌性,危害人体健康^[4⇓⇓-7]^。2009年和2019年全氟烷基磺酸(PFOS)和全氟辛酸(PFOA)及其盐类分别被列入斯德哥尔摩公约,160多个国家和地区同意减少并最终禁止使用该类物质^[8⇓⇓-11]^。随着长链全氟烷基羧酸类(≥C8)和全氟烷基磺酸类(≥C6)产品的消减和淘汰,大量短链全氟烷基羧酸类(<C8)和全氟烷基磺酸类(<C6)替代产品被开发和使用^[12]^。近年来研究发现短链PFASs在生物体中生物利用度高,大量使用可能产生同样的环境问题和健康风险。

PFASs可通过灰尘、饮用水以及动植物食物链等多种途径进入人体,其中摄食是人体暴露的最主要途径,研究报道78.9%的PFOS暴露来自水产品摄入^[13,14]^。中华绒螯蟹,俗称大闸蟹,是我国价值最高的水产品之一,味道鲜美,营养丰富,深受消费者的喜爱^[15,16]^。大闸蟹因底栖的生活模式、滤食的食性、移动性差等自身特点,极易摄入环境中的污染物,引起食品安全问题^[17,18]^。周殿芳等^[19]^在长江流域10个城市的河蟹中广泛检出PFASs,且总PFASs含量远高于其他水产品。鉴于短链和长链PFASs的危害性,亟待建立操作简便、快速高效的大闸蟹中短链和长链PFASs(C4~C14)同时检测的方法。

样品前处理方法包括萃取、净化和浓缩的过程,可以通过选择性富集目标物或净化提取液基质杂质,降低基质效应,特别是对于水产品这种基质复杂、PFASs痕量存在的生物样品,更需要选择合适的萃取方法来提高萃取效率和分析灵敏度^[20]^。目前常用的前处理方法主要有液液萃取、离子对液液萃取、液固萃取、消解法、液相微萃取、蛋白沉降技术等,其中液固萃取提取目标物种类多且效率较高,应用最为广泛^[21]^。水产样品基质复杂,需采用预处理技术进行净化以减少基质效应(matrix effect, ME),已有净化技术有固相萃取(SPE)、分散固相萃取、固相微萃取、QuEChERS等^[22⇓⇓-25]^。一般来说,SPE的重复性和精密度要优于其他3种萃取技术,更适用于水产品中痕量PFASs残留的准确定量检测。Groffen等^[24]^利用弱阴离子交换固相萃取柱净化的方法测定水产品中15种PFASs。柳思帆等^[26]^采用SPE前处理与HPLC-MS/MS相结合的方法分析测定了鱼样品中12种PFASs。但该类小柱需经活化、上样、淋洗和洗脱4个程序,操作繁琐、费时。相比于上述使用的SPE净化方法,快速滤过型净化柱(multi-plug filtration cleanup, m-PFC)方法具有操作简便、净化效率高的优点,在农药残留领域得到广泛应用^[27⇓-29]^。有学者基于m-PFC净化技术开发出蔬菜、畜禽产品中全(多)氟烷基化合物等多种污染物的快速检测方法^[30,31]^,但未见采用m-PFC净化方法检测大闸蟹中PFASs的报道。与其他水产品样本相比,大闸蟹样品中富含色素及油脂杂质,净化技术要求更高。该方法是基于吸附剂的萃取方法,吸附材料的性能直接制约着方法的萃取行为,碳纳米材料如石墨烯、氧化石墨烯和碳纳米管因具有比表面积大、可调控性强等优点被引入m-PFCs技术中^[32]^。已有研究发现羧基多壁碳纳米管具有对油脂等杂质吸附能力强但对PFASs吸附能力弱的特点,有望使用羧基多壁碳纳米管填料实现大闸蟹中杂质与PFASs的快速分离^[33]^。因此本研究以14种短链和长链PFASs(C4~C14)为目标物,通过优化前处理条件,开发了基于羧基多壁碳纳米管填料的快速滤过型净化柱,实现了大闸蟹中杂质与目标物质的一步分离,建立了大闸蟹中14种PFASs的快速分析技术,为大闸蟹中PFASs的批量快速检测提供了技术支撑。

## 1 实验部分

### 1.1 仪器与试剂

ACQUITY UPLC超高效液相色谱仪(美国Waters公司); AB 5500型三重四极杆质谱仪(美国AB SCIEX公司); MX-S涡旋仪(美国赛洛捷克公司); C-18R冷冻离心机(德国纳赫特公司); Milli-Q超纯水仪(美国Millipore公司); N-EVAP-12氮吹仪(美国Organomation公司)。

100 mg/L的全氟丁酸(PFBA)、全氟戊酸(PFPeA)、全氟己酸(PFHxA)、全氟庚酸(PFHpA)、全氟辛酸、全氟壬酸(PFNA)、全氟癸酸(PFDA)、全氟十一酸(PFUnDA)、全氟十二酸(PFDoDA)、全氟辛烷磺酸标准液以及50 mg/L的全氟十四酸(PFTeDA)、全氟癸烷磺酸(PFDS)标准液购自Wellington公司(圭尔夫,加拿大); 99.7%(纯度)的全氟丁基磺酸(PFBS)和全氟己烷磺酸(PFHxS)标准品购自Dr. Ehrenstorfer公司(奥格斯堡,德国); 2 mg/L的9种全氟烷基化合物同位素标记内标混合标准液(编号: MPFAC-MAX, 含^13^C_4_-PFBA、^13^C_2_-PFHxA、^13^C_4_-PFOA、^13^C_5_-PFNA、^13^C_2_-PFDA、^13^C_2_-PFUnDA、^13^C_8_-PFDoDA、^13^C_4_-PFHxS、^13^C_4_-PFOS)购自Wellington公司(圭尔夫,加拿大)。

色谱级甲醇(纯度99.9%)、色谱级乙腈(纯度99.9%)、甲酸(纯度99%)购自Merck(达姆施塔特,德国);醋酸铵(纯度99%,上海阿拉丁有限公司);氯化钠(分析纯,上海凌峰化学试剂有限公司);无水硫酸钠(分析纯,上海源叶生物科技有限公司);羧基多壁碳纳米管(纯度>95%,内径为3~5 nm,外径为8~15 nm,长度为50 μm)购自上海阿拉丁有限公司;十八烷基键合硅胶(C18,40~60 μm)购自上海怀聪生物科技有限公司。

样品购自上海青浦、崇明、松江中华绒螯蟹养殖基地,每份样品随机采集10只大闸蟹(100~150 g/只),雌雄各半。

### 1.2 标准溶液的配制

全氟烷基化合物标准贮备液:将购买的14种PFASs单标用甲醇稀释,配制为5 mg/L的标准贮备液,-18 ℃保存;同位素标记内标标准使用液:将购买的内标混合标准液用甲醇稀释为500 μg/L,作为内标使用液,-4 ℃保存。使用时恢复至室温,并摇匀。准确吸取适量的贮备液和20 μL同位素标记内标标准使用液,用甲醇稀释定容至1 mL,配成0.1~100 μg/L PFASs标准系列溶液(其中同位素内标质量浓度为10 μg/L)。

### 1.3 实验方法

#### 1.3.1 干扰与消除

为了降低背景值,实验过程中应避免使用聚四氟乙烯材质的色谱管路与器皿,本实验采用的色谱管路为聚醚醚酮(Peek)塑料管,样品小瓶为聚丙烯材质。

#### 1.3.2 样品前处理

将新鲜的大闸蟹样品洗净,解剖,取出可食用组织,冷冻干燥并记录含水量,研磨成粉,混匀后装入样品铝箔袋中冷冻保存,待分析用。准确称取0.5 g(精确至0.001 g)冷冻干燥后的大闸蟹样品放入50 mL聚丙烯离心管中,加入20 μL 500 μg/L同位素标记内标标准使用液,混匀后加入5 mL 0.1%甲酸水溶液,涡旋混匀,接着加入15 mL乙腈,加入萃取盐(2 g无水硫酸钠和2 g氯化钠),涡旋振荡3 min,以4000 r/min离心5 min,上清液待净化。

利用5 mL注射器制备简易快速滤过型净化柱,在柱管出液端安装一片下筛板,再将300 mg C18、100 mg羧基多壁碳纳米管充分混匀,从进液口端装填至柱管中,接着将上筛板安装入柱管内,利用转换接头与5 mL增压注射器连接。净化柱无需活化,取10 mL待净化上清液加载到滤过式净化柱上,用15 mL聚丙烯离心管收集全部流出液并经40 ℃水浴加热氮吹至近干,用甲醇超声复溶至1.0 mL,过0.22 μm尼龙有机相滤膜后,供UPLC-MS/MS分析。

### 1.4 仪器条件

超高效液相色谱条件 Shimadzu Shim-pack G1ST-C18色谱柱(100 mm×2.1 mm, 2 μm);流动相:(A)甲醇,(B)5 mmol/L乙酸铵溶液;流速:0.3 mL/min;柱温:40 ℃;进样体积:5 μL。梯度洗脱程序:0~0.5 min, 10%A~35%A; 0.5~3 min, 35%A~60%A; 3~5 min, 60%A~100%A; 5~6.5 min, 100%A; 6.5~7 min, 100%A~10%A。

质谱条件 电喷雾离子源(ESI),负离子模式;多反应监测(MRM)扫描;气帘气压力: 241.325 kPa(35.0 psi);喷雾电压: -4500 V;雾化温度: 500 ℃;雾化气压力: 344.750 kPa(50 psi);辅助气压力: 413.700 kPa(60 psi);去簇电压: 80 V。其他质谱参数见表1。

**表1:** 14种全氟烷基化合物及9种内标物的保留时间和质谱参数

No.	Compound name	Acronym	*t*_R_/ min	*M*_r_	Precursor ion (*m/z*)	Product ions (*m/z*)	CEs/eV	Corresponding internal standard
1	perfluorobutanoic acid	PFBA	2.81	214.04	212.9	168.8^*^	11	^13^C_4_-PFBA
2	perfluoro-*n*-pentanoic acid	PFPeA	3.32	264.05	262.7	218.9^*^, 174.9	15, 15	^13^C_4_-PFBA
3	perfluorohexanoic acid	PFHxA	3.63	314.05	312.9	268.8^*^, 118.9	11, 18	^13^C_2_-PFHxA
4	perfluoroheptanoic acid	PFHpA	3.87	364.06	363.1	318.9^*^, 168.9	15, 15	^13^C_2_-PFHxA
5	perfluorooctanoic acid	PFOA	4.09	414.07	413.4	368.9^*^, 168.9	13, 21	^13^C_4_-PFOA
6	perfluorononanoic acid	PFNA	4.28	464.08	462.5	419.0^*^, 168.9	16, 15	^13^C_5_-PFNA
7	perluorodecanoic acid	PFDA	4.50	514.08	512.8	468.8^*^, 268.9	14, 16	^13^C_2_-PFDA
8	perfluoroundecanoic acid	PFUnDA	4.72	564.09	562.7	518.9^*^, 268.9	13, 21	^13^C_2_-PFUnDA
9	perfluorododecanoic acid	PFDoDA	4.96	614.10	612.7	568.7^*^, 268.9	15, 25	^13^C_8_-PFDoDA
10	perfluorotetradecanoic acid	PFTeDA	5.48	714.11	712.9	668.7^*^, 168.9	15, 31	^13^C_8_-PFDoDA
11	perfluoro-1-butane sulfonic acid	PFBS	3.34	300.19	299.3	80.0^*^, 99.0	33, 42	^13^C_4_-PFHxS
12	perfluoro-1-hexane sulfonic acid	PFHxS	3.86	400.20	399.3	80.0^*^, 99.0	40, 35	^13^C_4_-PFHxS
13	perfluoro-1-octane sulfonic acid	PFOS	4.25	500.22	499.2	99.0^*^, 80.0	64, 34	^13^C_4_-PFOS
14	perfluoro-1-decanesulfonate	PFDS	4.69	600.22	599.2	80.0^*^, 99.0	50, 45	^13^C_4_-PFOS
15	perfluoro-*n*-[^13^C_4_] butanoicacid	^13^C_4_-PFBA	2.81	218.04	217.0	172.0^*^	11	-
16	perfluoro-*n*-[1,2-^13^C_2_] hexanoicacid	^13^C_2-_PFHxA	3.63	316.05	315.0	270.0^*^	11	-
17	perfluoro-*n*-[1,2,3,4-^13^C_4_] octanoicacid	^13^C_4_-PFOA	4.09	418.07	417.0	372.0^*^	13	-
18	perfluoro-*n*-[1,2,3,4,5-^13^C_5_] octanoic acid	^13^C_5_-PFNA	4.28	469.08	468.0	423.0^*^	16	-
19	perfluoro-*n*-[1,2-^13^C_2_] decanoicacid	^13^C_2_-PFDA	4.50	516.08	515.0	470.0^*^	14	-
20	perfluoro-*n*-[1,2-^13^C_2_] undecanoicacid	^13^C_2_-PFUnDA	4.72	566.09	565.0	520.0^*^	13	-
21	perfluoro-*n*-[1,2-^13^C_8_] dodecanoicacid	^13^C_8_-PFDoDA	4.96	616.10	615.0	570.0^*^	15	-
22	sodiumperfluoro-1-hexane[^13^C_4_] sulfonate	^13^C_4_-PFHxS	3.86	404.20	402.9	103.0^*^	35	-
23	perfluoro-1-[1,2,3,4-^13^C_4_] octane-sulfonate	^13^C_4_-PFOS	4.25	504.22	502.9	99.0^*^	34	-

* Quantitative ion. CEs: collision energies.

## 2 结果与讨论

### 2.1 色谱和质谱条件优化

据文献[34⇓-36]报道,甲醇作为流动相中的有机相对目标物质的分离度优于乙腈,在水相中加入酸能够保持一定的pH值及离子强度,减少拖尾,改变峰形。因此,本实验比较了5 mmol/L草酸水溶液-甲醇、0.1%甲酸水溶液-甲醇、5 mmol/L乙酸铵水溶液-甲醇、50 mmol/L乙酸铵水溶液-甲醇作为流动相的效果。结果表明,水相中加入5 mmol/L乙酸铵能够显著改变峰形,乙酸铵增加到50 mmol/L时,峰形未得到改变。因此选择5 mmol/L乙酸铵水溶液-甲醇为流动相体系,14种PFASs的提取离子色谱图见图1。

**图1:**
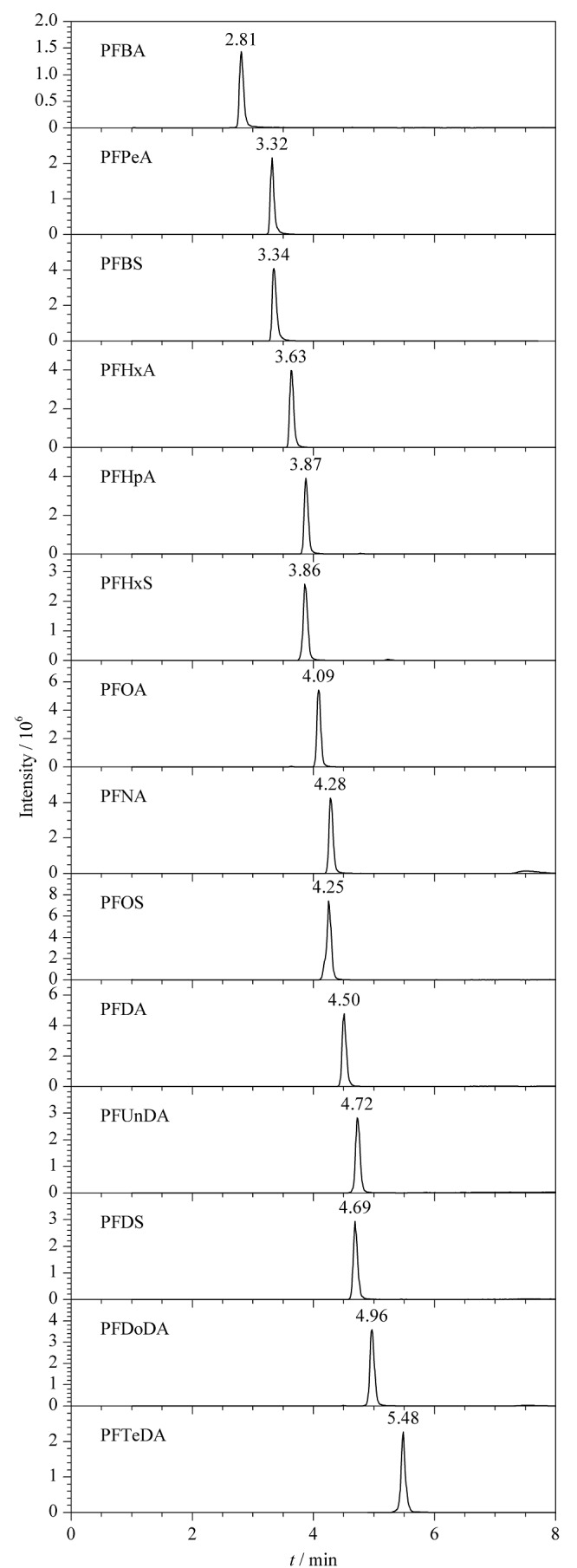
14种PFASs混合标准溶液(50 μg/L)的提取离子色谱图

PFASs难以质子化,因此采用电喷雾离子源负离子模式。采用针泵以20 μL/min的速度将100 μg/L的14种PFASs目标物标准溶液注入离子源中,在MRM模式下对各目标物分别进行一级质谱分析(Q1扫描)和二级质谱分析(Q3扫描),优化碰撞能量等参数,使离子对的响应达到最大,各物质的最优质谱参数见表1。

### 2.2 提取条件优化

#### 2.2.1 提取溶剂优化

14种PFASs碳链长度跨度大,理化性质差异大,lg *K*_ow_值随着碳链的增加而增加(如全氟丁酸为2.43,全氟癸酸为7.90)^[36]^。因此提取溶剂的选择和优化需充分考虑溶剂极性、酸碱度以及基质特点。实验以阴性加标样品(20 μg/kg)为对象,采用外标法比较了乙腈、甲醇、10 mmol/L NaOH乙腈、1%甲酸乙腈、乙腈-水(9∶1, v/v)、乙腈-1%甲酸水溶液(9∶1, v/v)、乙腈-0.1 mmol/L草酸水溶液(9∶1, v/v)7种常用提取溶剂的提取效率。如图2a所示,不同提取溶剂对14种全氟烷基化合物提取效果存在显著差异。甲醇提取14种目标物的回收率为12%~39%,乙腈提取的回收率为8%~51%。乙腈对4种全氟磺酸和长链全氟羧酸(C12、C14)的提取效率优于甲醇。与乙腈相比,酸化乙腈(1%甲酸乙腈)未对目标物提取效率产生明显的影响。乙腈-水(9∶1, v/v)对14种目标物的提取效率较纯乙腈提升了9%~85%。

**图2:**
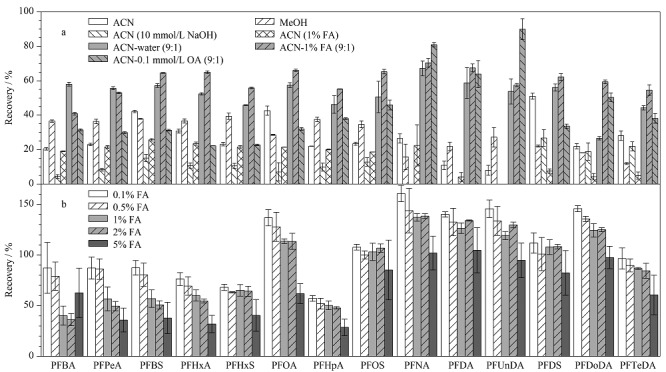
(a)提取溶剂和(b)甲酸体积分数对14种PFASs提取回收率的影响(*n*=3)

PFASs与生物基质以离子形式结合,改变提取剂的酸度避免酸性目标物离子化,可适当提高萃取效率^[37,38]^。在乙腈中加入1%甲酸水溶液时,除短链的全氟丁酸和全氟戊酸外,其余12种目标物的回收率较乙腈水体系增加了5%~123%;加入0.1 mmol/L草酸水溶液仅提高了全氟辛烷磺酸和全氟十一酸的提取率。

同时考察了不同甲酸水溶液中甲酸体积分数(0.1%、0.5%、1%、2%、5%)对14种PFASs提取效率的影响。结果如图2b所示,14种PFASs的提取效率随着甲酸水溶液中甲酸含量的增加而不断降低,这与前人报道的结果^[31]^一致。表明在合适的酸性条件下目标物更容易被有机溶剂提取。综上,选择乙腈-0.1%甲酸水溶液作为提取溶剂进一步优化提取条件。

#### 2.2.2 提前加入0.1%甲酸水溶液体积、提取时间及萃取盐优化

与纯乙腈相比,在乙腈中加入0.1%甲酸水溶液后目标化合物的提取效率显著提升,猜想在提取过程中提前加入不同体积0.1%甲酸水溶液浸润样品可能影响14种PFASs的提取效率。因此研究了提前加入不同体积(1.5、5、8、10、15 mL)的0.1%甲酸水溶液对14种PFASs提取效率的影响。结果如图3a所示,0.1%甲酸水溶液的体积显著影响短链全氟烷基羧酸类物质(PFBA、PFPeA、PFHxA)的提取效率,如加入1.5 mL 0.1%甲酸水溶液提取,PFHxA的提取效率为77.1%,加入5 mL 0.1%甲酸水溶液提取时PFHxA的提取效率达到最大,为91.2%,加入15 mL 0.1%甲酸水溶液提取时PFHxA的提取效率降为86.3%。表明加入合适体积的0.1%甲酸水溶液浸润样品,能够增加提取溶剂和目标物的接触,提升目标物的提取效率^[26,31]^。因此选择加入 0.1%甲酸水溶液5 mL。

**图3:**
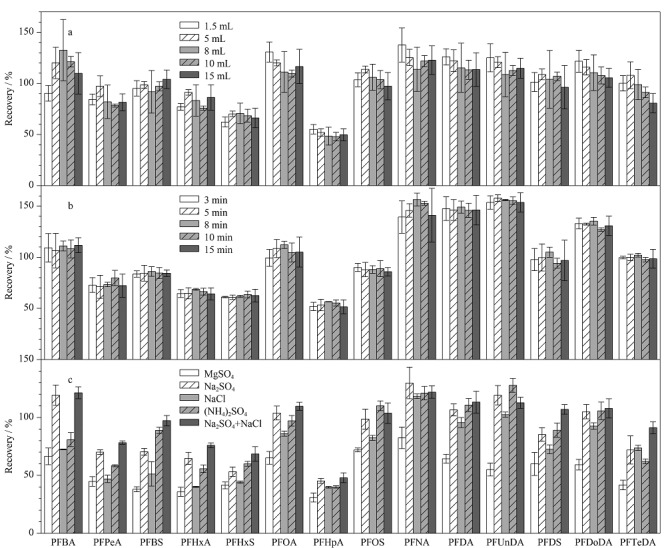
(a)0.1%甲酸水溶液体积、(b)提取时间、(c)萃取盐类型对14种PFASs 提取回收率的影响(*n*=3)

涡旋提取时间及萃取盐种类也可能影响14种PFASs的提取效率。考察了不同涡旋提取时间(3、5、8、10、15 min)及不同萃取盐(MgSO_4_、Na_2_SO_4_、NaCl、(NH_4_)_2_SO_4_、Na_2_SO_4_+NaCl)对14种PFASs提取效率的影响。涡旋提取时间在3~15 min内,14种PFASs的提取效率未随提取时间变化产生明显差异(图3b),表明涡旋提取时间不是影响14种PFASs提取效率的主要因素,实验最终选择涡旋提取3 min。5种萃取盐显著影响14种PFASs的提取效率,MgSO_4_作为萃取盐时14种PFASs的提取效率最低(30.9%~82.3%), Na_2_SO_4_+NaCl作为萃取盐时,14种PFASs的提取效率最高,为47.9%~121.9%(图3c)。研究表明在样品前处理过程加入Na_2_SO_4_可以去除提取液中的水分,使浓缩复溶后的样品溶液体积更准确,同时加入NaCl有利于提取溶剂和水相分层,防止样品中的水分和水溶性极性基质干扰物进入提取液中,从而减少对目标物的干扰和质谱离子源的污染^[39]^。因此,综合考虑时间成本、经济成本,选择提前添加5 mL 0.1%甲酸水溶液和Na_2_SO_4_、NaCl,涡旋提取3 min。

### 2.3 快速滤过型净化方法优化

对于滤过型净化方式,减少上样量可以有效降低复杂基质的基质效应,但也存在回收率和灵敏度降低的缺陷,寻找基质效应和回收率的平衡点是提高检测方法灵敏度的关键^[24]^。因此以基质效应、回收率为指标,考察了不同体积(5、10、13 mL)的提取液过羧基多壁碳纳米管滤过型净化柱的净化效果。以基质匹配标准溶液峰面积与空白溶剂标准溶液峰面积的比值计算基质效应。如图4所示,不同提取液体积过柱后,除PFBA、PFPeA外,其余12种目标物的基质抑制效应随过柱液体积的增加而增加。随过柱液体积增加大部分目标物质回收率逐渐增加。尤其短链全氟羧酸类物质(PFBA、PFPeA和PFHxA), 5 mL提取液过柱情况下3种短链同系物回收率均低于50%,随着上样量增加回收率提升到65%以上。表明减少过柱液体积能够不同程度的减弱基质抑制效应,但较少的过柱液体积使溶液在净化填料中留存,造成回收率降低。综合基质效应、回收率以及灵敏度,最终选择10 mL提取液过净化柱。在10 mL提取液过净化柱条件下,14种PFASs的基质效应为32.9%~101%,除PFDS外,其余13种目标物均表现出不同程度的基质抑制效应,因此本实验采用内标法以降低基质效应对目标化合物的影响。

**图4:**
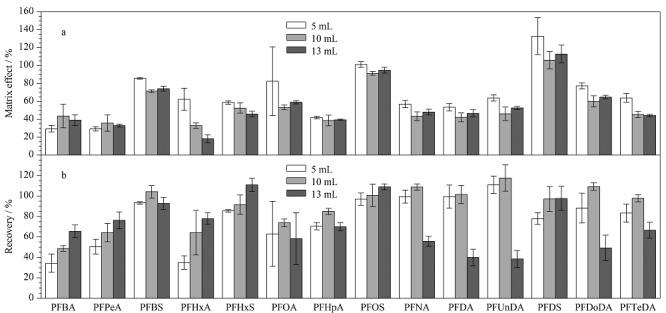
上样体积对14种PFASs(a)基质效应和(b)回收率的影响(*n*=3)

### 2.4 方法学指标

#### 2.4.1 线性范围与检出限

采用配制的PFASs标准系列溶液,以内标法进行定量分析,在最优的仪器条件下进行测定。以各待测物和内标物的色谱峰面积比值为纵坐标、对应的质量浓度为横坐标进行线性回归分析,绘制标准曲线。方法的线性方程、线性范围及相关系数(*R*^2^)见表2。

**表2:** 14种PFASs的线性方程、线性范围、检出限和定量限

Compound	Linear equation	Linear range/(μg/L)	*R*^2^	LOD/(μg/kg)	LOQ/(μg/kg)
PFBA	*y*=0.41*x*+0.28	0.50-100	0.999	0.15	0.50
PFPeA	*y*=0.64*x*+0.094	0.50-100	0.999	0.15	0.50
PFBS	*y*=6.69*x*+0.54	0.10-100	0.998	0.03	0.10
PFHxA	*y*=0.42*x*+0.039	0.10-100	0.998	0.03	0.10
PFHpA	*y*=0.56*x*+0.087	0.10-100	0.998	0.03	0.10
PFHxS	*y*=1.97*x*+0.052	0.10-100	0.999	0.03	0.10
PFOA	*y*=0.69*x*+0.047	0.50-100	0.999	0.15	0.50
PFOS	*y*=1.20*x*+0.024	0.50-100	0.999	0.15	0.50
PFNA	*y*=0.81*x*+0.12	0.50-100	0.999	0.15	0.50
PFDA	*y*=0.57*x*+0.076	0.10-100	0.998	0.03	0.10
PFUnDA	*y*=0.88*x*+0.090	0.50-100	0.998	0.15	0.50
PFDS	*y*=0.27*x*+0.0010	0.50-100	0.999	0.15	0.50
PFDoDA	*y*=0.57*x*+0.062	0.10-100	0.999	0.03	0.10
PFTeDA	*y*=0.086*x*+0.0038	0.10-100	0.999	0.03	0.10

*R*^2^: correlation coefficient; *y*: chromatographic peak area ratio of each target compound to corresponding internal standard; *x*: mass concentration of target compound, μg/L.

由表2可以看出,PFBS、PFHxA、PFHpA、PFHxS、PFDA、PFDoDA、PFTeDA在0.10~100 μg/L范围内,其余7种PFASs在0.50~100 μg/L内呈现较好的线性关系,*R*^2^为0.998~0.999。以信噪比(*S/N*)=3和*S/N*=10分别确定各目标物的检出限(LOD)和定量限(LOQ)。14种全氟烷基化合物的检出限和定量限分别为0.03~0.15 μg/kg和0.10~0.50 μg/kg,满足痕量检测要求。

#### 2.4.2 回收率和精密度

在大闸蟹组织样品中设置低(方法定量限)、中、高3个添加水平,每个水平重复6次。通过内标法定量,同时做基质空白和系统空白试验,扣除实验本底值后计算加标回收率和相对标准偏差(RSD)。结果如表3所示。在加标浓度范围内,14种全氟烷基化合物的平均回收率为73.1%~120%, RSD为1.68%~19.5%,符合GB/T 27404-2008附录中的回收率、精密度标准结果要求,表明方法具有良好的准确性和重复性。该检测方法与现行国家标准和文献报道的动物源食品基质中PFASs测定方法的指标基本一致^[38,40,41]^,但净化时间由传统方法的30 min缩短至数十秒,大大提高了分析效率。

**表3:** 大闸蟹样品中14种PFASs的加标回收率和相对标准偏差(*n*=6)

Compound	Spiked level/(μg/kg)	Recovery/%	RSD/%	Compound	Spiked level/(μg/kg)	Recovery/%	RSD/%
PFBA	0.50	98.0	9.91	PFOS	0.50	75.5	16.8
	3.0	93.7	17.7		3.0	86.0	19.2
	5.0	100	2.10		5.0	79.2	3.01
PFPeA	0.50	93.0	11.8	PFNA	0.50	84.2	19.6
	3.0	109	12.0		3.0	110	17.9
	5.0	95.8	8.72		5.0	92.8	1.68
PFBS	0.10	100	11.1	PFDA	0.10	105.4	9.10
	0.50	97.3	12.2		0.50	81.9	15.3
	5.0	105	2.55		5.0	95.1	5.14
PFHxA	0.10	87.1	8.32	PFDS	0.50	96.5	8.86
	0.50	95.4	9.22		3.0	120	13.7
	5.0	82.0	3.48		5.0	110	13.6
PFHpA	0.10	87.1	8.32	PFUnDA	0.50	104	13.6
	0.50	86.2	8.93		3.0	87.8	19.3
	5.0	73.1	4.90		5.0	96.6	3.45
PFHxS	0.10	93.4	9.89	PFDoDA	0.10	112	13.6
	0.50	89.8	5.36		0.50	116	13.0
	5.0	82.1	2.80		5.0	120	6.98
PFOA	0.50	109	19.5	PFTeDA	0.10	98.8	16.2
	3.0	97.2	1.71		0.50	77.8	11.4
	5.0	96.0	2.70		5.0	79.6	5.18

### 2.5 实际样品检测

随机采集了上海青浦、松江、崇明3个养殖基地的9份中华绒螯蟹样品,按照本研究建立的方法进行检测,结果见表4。由结果可以看出,除PFBA、PFPeA、PFHxA、PFHpA、PFBS和PFDS 6种物质外,其余8种物质全部可以检出,其中PFDA、PFUnDA、PFDoDA、PFTeDA、PFOS的检出率高达100%。14种PFASs的总含量为3.52~37.77 μg/kg,平均值为14.11 μg/kg。PFDA、PFUnDA、PFOS、PFDoDA为主要污染同系物,分别占14种全氟烷基化合物总量的31.2%、30.6%、15.0%、10.9%。受管控的PFOS平均含量为2.26 μg/kg,远低于欧盟针对生物体中PFOS的环境质量标准限量^[42]^。实验结果表明,碳链长度为8~12的全氟羧酸类同系物和PFOS更易在大闸蟹可食用组织中蓄积,但实验样本数量较少,有必要进一步评估大闸蟹中的PFASs暴露风险。

**表4:** 不同大闸蟹样品中14种PFASs的含量及检出率

Compound	Contents in different samples/(μg/kg)													Detection rate/%
	1	2	3	4	5	6	7	8	9	Maximum	Minimum	Median	Average	
PFBA	-	-	-	-	-	-	-	-	-	-	-	-	-	0
PFPeA	-	-	-	-	-	-	-	-	-	-	-	-	-	0
PFHxA	-	-	-	-	-	-	-	-	-	-	-	-	-	0
PFHpA	-	-	-	-	-	-	-	-	-	-	-	-	-	0
PFOA	0.81	0.39	-	-	-	0.16	-	0.52	-	0.81	-	-	0.21	44.44
PFNA	-	-	-	-	0.54	-	-	0.61	-	0.61	-	-	0.13	22.22
PFDA	8.15	1.87	2.13	0.85	15.71	1.02	1.87	13.66	1.52	15.71	0.85	1.87	5.20	100.00
PFUnDA	5.60	1.90	2.06	1.25	11.57	1.20	1.93	12.27	2.01	12.27	1.20	2.01	4.42	100.00
PFDoDA	1.23	0.71	0.69	0.77	2.72	0.54	0.44	2.90	0.66	2.90	0.44	0.71	1.18	100.00
PFTeDA	0.63	0.53	0.56	0.36	0.79	0.51	0.50	0.69	0.39	0.79	0.36	0.53	0.55	100.00
PFBS	-	-	-	-	-	-	-	-	-	-	-	-	-	0
PFHxS	0.14	-	-	-	0.88	-	-	0.52	-	0.88	-	-	0.17	33.33
PFOS	3.70	1.23	0.88	0.30	5.58	0.47	1.25	6.14	0.77	6.14	0.30	1.23	2.26	100.00
PFDS	-	-	-	-	-	-	-	-	-	-	-	-	-	0
∑PFASs	20.25	6.63	6.32	3.52	37.77	3.89	5.98	37.31	5.35	37.77	3.52	6.32	14.11	

-: not detected.

## 3 结论

本文建立了基于羧基多壁碳纳米管的快速滤过型净化柱结合超高效液相色谱-串联质谱快速检测大闸蟹样品中14种PFASs的方法。经考察,本法精密度、准确度和灵敏度均满足检测要求。本方法克服了传统方法步骤繁琐、耗时长的缺点,实现了操作简便、快速高效的目标,完全满足批量中华绒螯蟹中14种PFASs的快速检测。
